# Artificial intelligence for university physical education: a data–knowledge synergy digital-intelligent sports platform

**DOI:** 10.3389/fpubh.2026.1835590

**Published:** 2026-09-08

**Authors:** Jiacheng Feng, Meijie Yao, Xueying Feng, Chenrui Wang, Hongmiao Chen, Dong Li, Qimeng Niu

**Affiliations:** 1 Department of Physical Education, Tianjin University of Technology, Tianjin, China; 2 Physical Education Teaching and Research Department, Nanqiao Middle School, Jinjiang, China; 3 School of Innovation and Technology, The Glasgow School of Art, Glasgow, United Kingdom; 4 School of Electrical Engineering and Automation, Tianjin University of Technology, Tianjin, China; 5 College of Physical Education, Huaqiao University, Quanzhou, China; 6 School of Physical Education and Health, Zhaoqing University, Zhaoqing, China

**Keywords:** artificial intelligence, basic psychological needs, digital-intelligent sports, retrieval-augmented generation, university physical education

## Abstract

This study presents the design, construction, and preliminary evaluation of a university-oriented digital-intelligent sports platform integrating a large language model with retrieval-augmented generation (RAG), campus data services, and domain knowledge resources. The platform architecture comprises technical, knowledge, operational-data, and user-application components supporting teaching assistance, exercise guidance, competition organization, and health-related information services. A formative campus deployment involving 428 students and six instructors documented implementation feasibility and user-facing application scenarios. To provide a more rigorous empirical supplement, an independent six-week, non-randomized, intact-class quasi-experiment was conducted with 196 students in four physical-education classes. Two classes received platform-supported instruction and two received conventional instruction from the same instructor. Baseline-adjusted analyses indicated higher posttest basic psychological need satisfaction in the platform-supported group (adjusted difference = 0.618, 95% CI 0.506–0.730, *p* < 0.001) and lower need frustration (adjusted difference = −0.653, 95% CI −0.759 to −0.547, *p* < 0.001). The adjusted difference in self-reported physical activity was not statistically significant (*p* = 0.216). These findings provide preliminary evidence of more favorable psychological-need experiences during platform-supported physical education, but do not indicate a corresponding increase in self-reported physical activity over 6 weeks. The central contribution lies in the education-oriented integration of AI architecture, institutional knowledge resources, and classroom implementation evidence within a unified university physical-education platform.

## 1 Introduction

Since 1985, when the National Health Commission and related ministries launched the National Survey on Students’ Physical Fitness and Health, longitudinal evidence has continued to document concerns regarding the physical fitness and cardiovascular risks of Chinese college students (1). Broader reviews have also identified declines in several fitness components among children and adolescents over time (2). Current university physical education is often characterized by uniform curricula, standardized evaluation, and insufficient personalization. Traditional transmission-based instruction may not fully satisfy the growing need for interaction and individualized learning, underscoring the need to move from standardized delivery toward more responsive teaching through technological innovation. Guided by national strategies for building a leading sports nation and an education power, AI provides an important technical and institutional context for reforming university physical education.

Artificial intelligence has extended traditional physical spaces into digital and networked environments. These intelligent spaces retain the pedagogical logic of conventional teaching while providing teachers and students with more timely and personalized services. In sports, AI applications have advanced rapidly: big-data analytics, motion recognition, virtual reality, and wearable technologies support hybrid teaching spaces, individualized guidance, and real-time performance monitoring (3–7). The integration of digital twins and multimodal technologies may further enhance interactivity and data-informed governance (8). Together, these developments demonstrate AI’s potential to extend management from classroom instruction to broader learning and health-related services, while also raising questions of technical validity, data governance, and teacher oversight (9).

Nevertheless, existing solutions for university sports and physical education still show clear structural limitations. Many domestic campus platforms prioritize administrative digitization (e.g., attendance, venue booking, and fitness-test reporting), which improves management efficiency but often leaves sport-science and PE knowledge fragmented and difficult to translate into actionable support for teaching and student development. In contrast, many international sport-technology products emphasize performance analytics and individualized monitoring, yet they are frequently designed for elite-athlete contexts or single-function scenarios (e.g., load tracking or technique feedback) rather than the holistic needs of higher education PE. As a result, there remains a practical gap for a university-oriented system that can connect campus data with credible pedagogical knowledge and serve teaching, participation, and health promotion in an integrated manner.

In this study, LLMs+RAG is adopted primarily to strengthen physical-education pedagogy in universities rather than to optimize competitive training. Retrieval-augmented generation is used to ground explanations and recommendations in retrieved institutional and professional sources (e.g., PE curricula, physical-fitness standards, health-promotion guidance, skill-learning materials, and campus activity policies), with the aim of providing more consistent support for core educational goals: improving students’ physical fitness and health literacy, encouraging sustained activity participation, fostering a positive campus sport atmosphere, and facilitating sport-skill learning through clear, level-appropriate explanations and formative feedback. By combining evidence retrieval with natural-language interaction, the system can help instructors and students interpret fitness-test results, understand skill points and common errors, plan safe and feasible activity routines, and access learning resources in a timely, inclusive, and understandable way—while keeping teachers in control of instructional decisions and ensuring that high-stakes guidance remains accountable. Retrieval can improve grounding in knowledge-intensive tasks, but it does not by itself guarantee factual correctness; source traceability and teacher review remain necessary (10, 11).

For the classroom evaluation, Basic Psychological Need Theory within self-determination theory provides a proximal educational criterion. Need satisfaction reflects experiences of autonomy, competence, and relatedness, whereas need frustration reflects pressure, ineffectiveness, and social exclusion (12, 13). In physical education, autonomy-supportive teaching and meaningful feedback are associated with more self-determined motivation and engagement (14–17). The present study uses these outcomes to supplement—not replace—the platform-construction analysis.

These components are treated as a connected socio-technical educational system: the technical architecture enables platform-supported teaching, while the classroom evaluation examines students’ proximal educational experiences within that environment. Accordingly, the study makes three connected contributions. First, it presents a university-oriented digital-intelligent sports platform that integrates teaching, extracurricular participation, competition services, and health-related support. Second, it introduces a data-knowledge synergy approach that couples structured campus data with retrieval-grounded domain knowledge through LLMs+RAG. Third, it links a real-world campus deployment with an independent six-week classroom evaluation, providing preliminary evidence of implementation feasibility and students’ psychological experiences during platform-supported instruction.

## 2 Design and construction of a digitally intelligent platform

As a vehicle for integrating digital technology with sport science, the digital-intelligent sports platform—built upon IoT-compatible data access, large language models (LLMs), and big-data analytics—provides a technical framework for modern sports services (4, 18, 19). The university-oriented platform developed in this study employs an LLM architecture enhanced by retrieval-augmented generation (RAG) and a proprietary knowledge architecture to address three practical challenges in traditional sports services: the conflict between standardized instruction and individual differences, the imbalance between immediacy and continuity in exercise intervention, and the fragmentation of health-management systems. By establishing a closed loop of data acquisition, intelligent analysis, personalized delivery, and effect evaluation, the platform aims to move from generalized services toward more individualized guidance.

**Figure 1:**
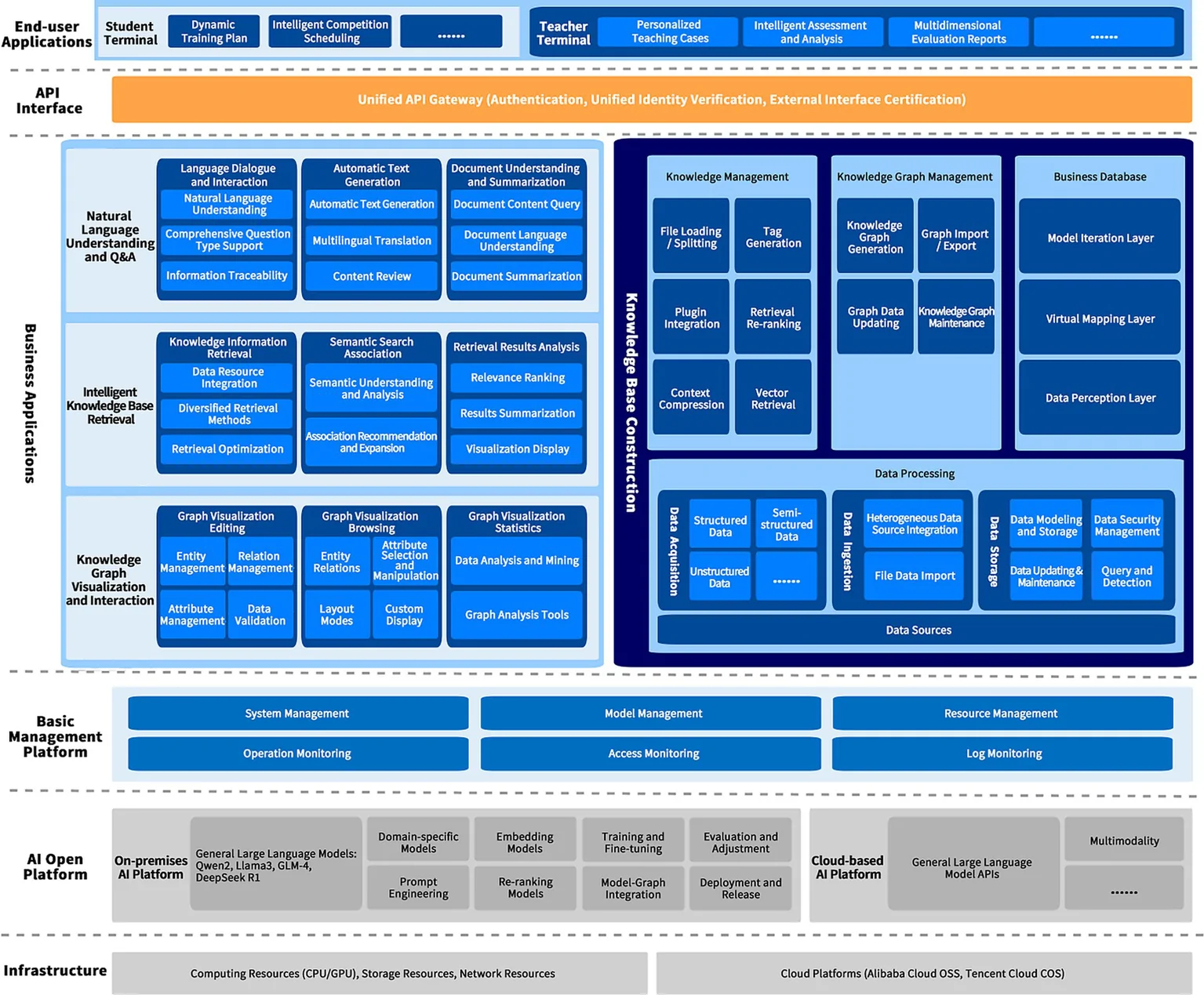
Digital-intelligent platform technology framework.

**Figure 2:**
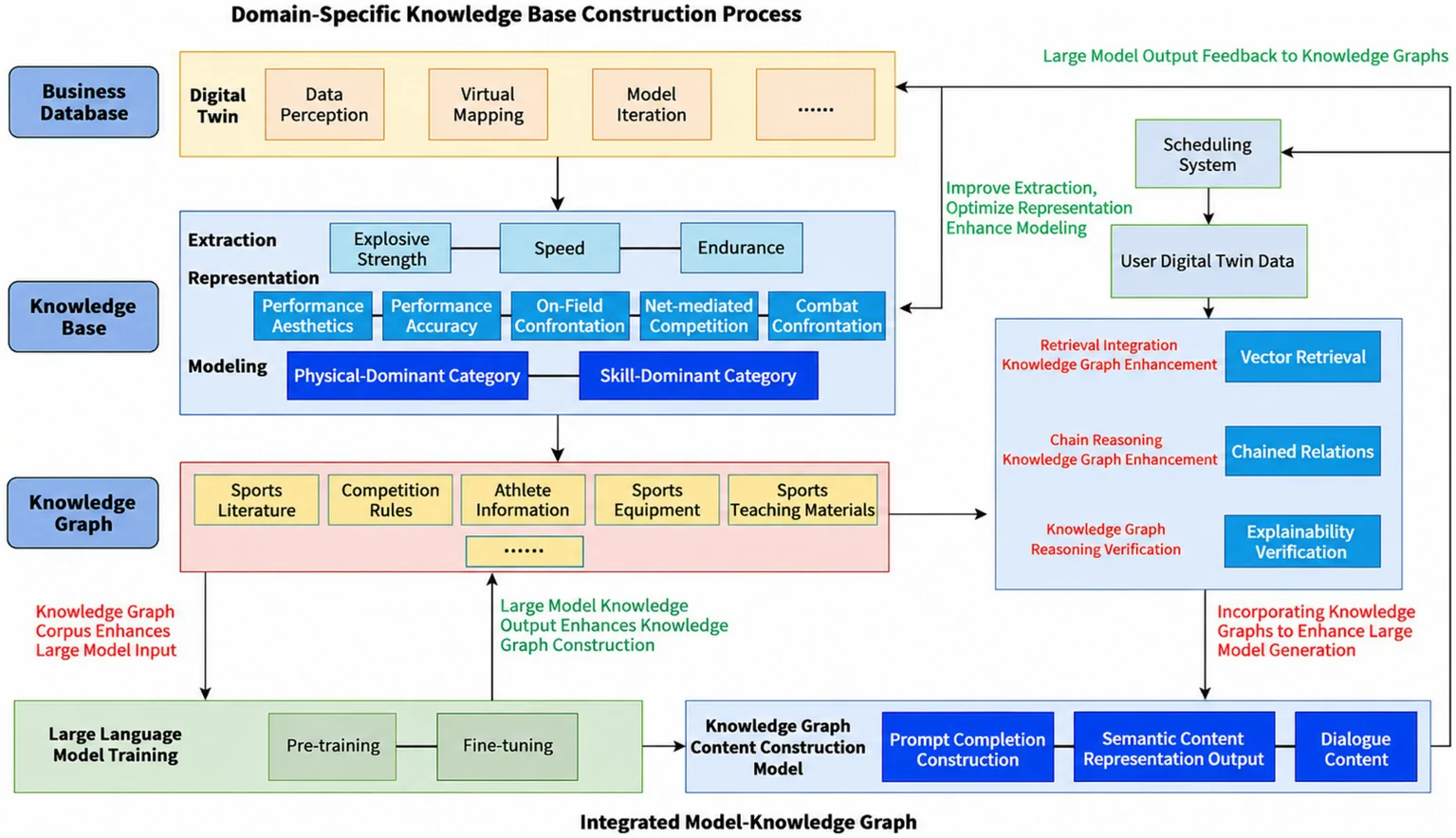
Proprietary knowledge-base architecture.

**Figure 3:**
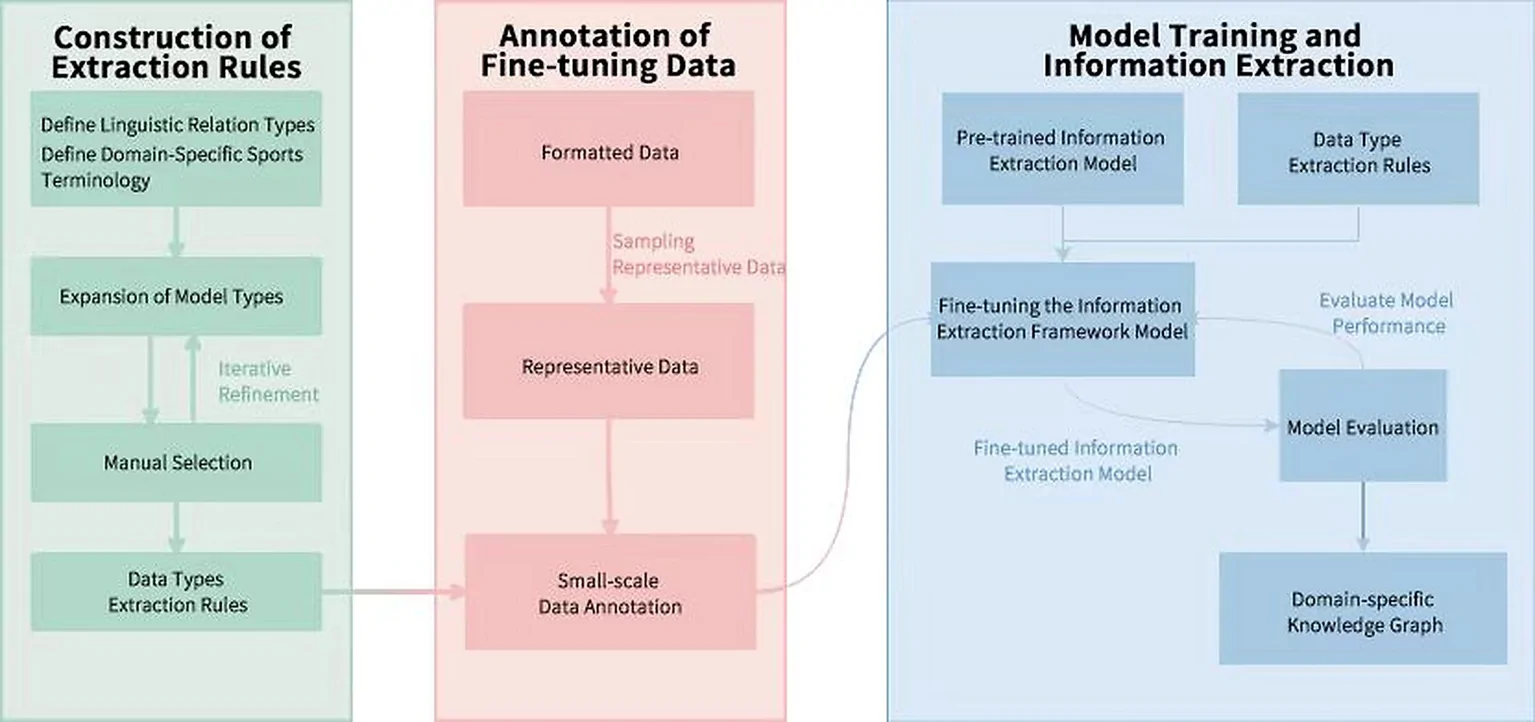
Knowledge-graph construction process.

**Figure 4:**
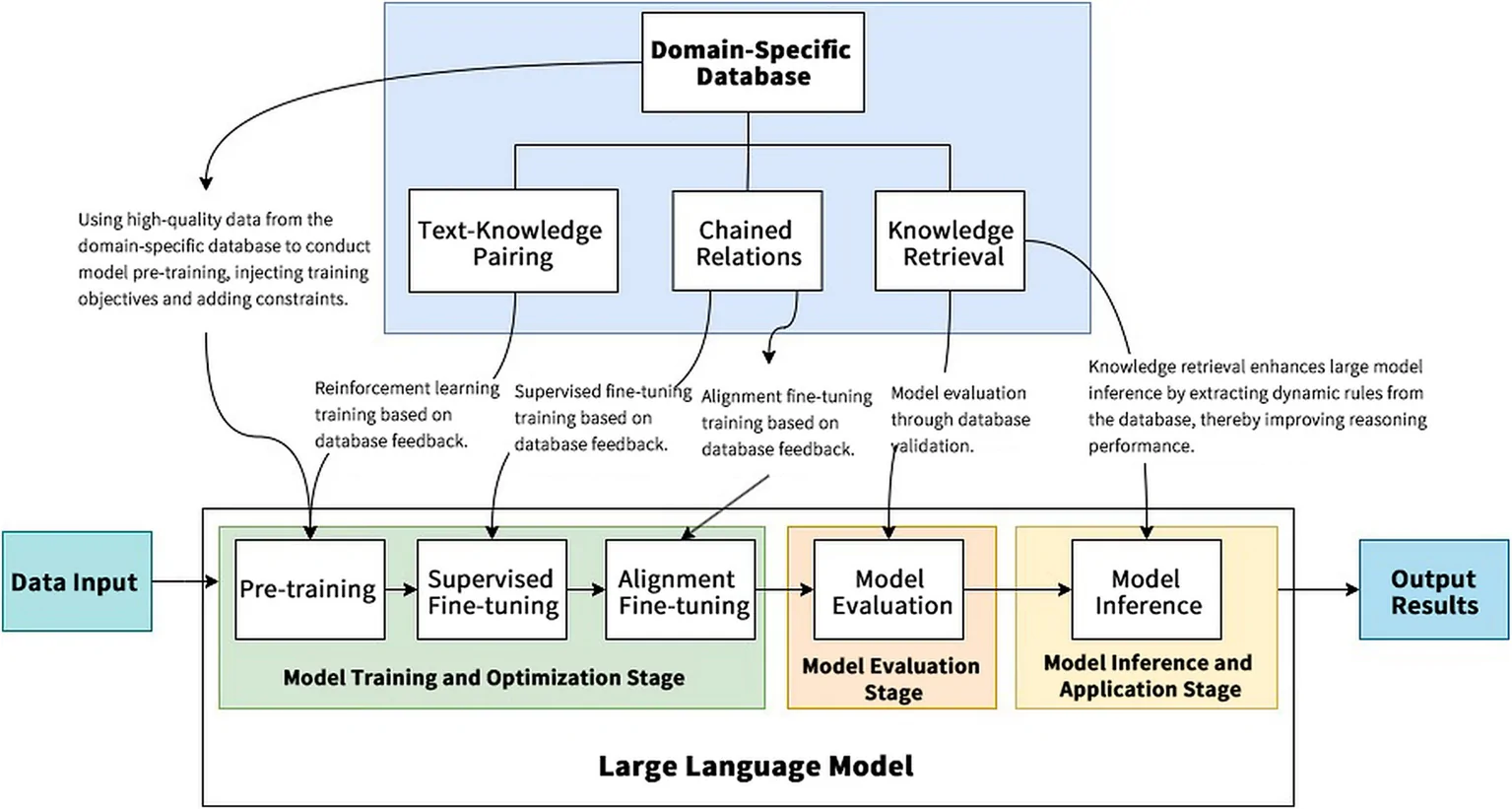
Knowledge-base construction and model interaction. Figures 1–4 summarize the integrated system architecture and module relationships applied during the university deployment.

Figures 1–4 present the integrated system architecture and the principal model, knowledge, database, and application components applied during the university deployment.

At the theoretical level, the system uses LLMs with second-stage retrieval from a proprietary knowledge architecture to improve evidence grounding and domain relevance rather than to guarantee error-free output (10, 11, 20). This architecture integrates three components: a knowledge graph, a sports knowledge repository, and a business (operational) database (21). The knowledge graph, constructed through LLM-assisted semantic parsing and expert verification, connects key entities in sport science; the knowledge repository organizes institutional and professional resources; and the operational database links authorized teaching and service information. A sports digital twin is retained as an architecture-level extension for dynamically mapping physiological and behavioral information (8). Collectively, these mechanisms provide a foundation for student-centered services and outcome evaluation.

At the practical level, the platform functions through three subsystems:

1. Teaching-assistance system—powered by LLMs and retrieved resources to support teacher-student interaction, question answering, and content organization.
2. Social-interaction system—built on sports resources and campus service data to support competition organization and achievement-based participation incentives. Token-like achievements are treated as non-speculative educational design concepts within the participation-support framework.
3. Healthy-living system—integrates authorized fitness information, exercise guidance, and venue services to support educational interpretation and teacher-supervised planning rather than medical diagnosis.

Technically, the platform’s architecture comprises three layers—foundation, platform, and application—corresponding, respectively, to technical framework design, proprietary knowledge-base construction, and user-module deployment. Its four-tier software stack—comprising front-end interfaces, business applications, AI management and service platforms, and underlying infrastructure—enables multimodal interaction, AI reasoning, and collaborative intelligence. Through this integration, the system supports intelligent instruction, personalized training guidance, and evidence-informed management in university sports, as shown in Figure 1.

### 2.1 Design of the technical framework

#### 2.1.1 User-side design: application scenarios for instructors and students

In the platform’s user-side design, teacher and student modules perform distinct yet complementary functions in physical education and training.

Teacher-side modules focus on instructional design, data analytics, and evaluation. The intelligent lesson-preparation system, powered by LLMs, generates revisable lesson-plan drafts, integrates historical teaching cases with the knowledge base, and embeds multimodal resources such as image demonstrations and 3D tactics-board sketches. Combined with structured reasoning prompts (22), it follows the logic of instructional objectives → student competencies → technical–tactical alignment, supporting closer matching between learners’ needs and training content, thereby improving instructional precision and scientific rigor. Generated materials remain subject to teacher verification.

The student-profiling and analytics module captures authorized physiological and behavioral data—such as heart rate, posture, task completion, and interaction frequency—and visualizes them for instructors to assess individual progress. IoT sensors and computer-vision methods provide technical routes for dynamic evaluation, while pose estimation and object detection support movement analysis (23–25). The intelligent assessment subsystem combines video analysis with LLM-generated semantic feedback to produce multidimensional reports, consistent with AI-supported evaluation approaches in university physical education (26).

On the student side, the platform offers personalized exercise-guidance and competition functions. The AI fitness coach, informed by each student’s available profile and goals, uses retrieved resources and adaptive recommendation rules to provide exercise suggestions and generated text or image prompts. Transfer learning and recommender methods provide technical routes for adapting content (27, 28). The competition module supports fixture generation, venue coordination, opponent matching, predictive assistance, and scheduling workflows under instructor review. A gamified interaction assistant provides virtual challenges and achievement records to support participation.

In university PE, digital achievement credentials are designed as educational incentives rather than speculative assets. Achievement records can be issued after platform-verified participation (e.g., attendance, venue check-in, or wearable-confirmed moderate-intensity activity duration) and mapped to PE objectives such as fitness improvement, skill mastery, and sustained participation. To avoid one-size-fits-all incentives, thresholds can be adapted to baseline fitness levels and course types (e.g., table tennis, basketball, and aerobics), with multiple recognition pathways for participation, improvement, teamwork, and health-literacy tasks. Such records are intended primarily for on-campus recognition, while teacher-defined safety constraints prevent incentives that might encourage excessive load or risky behaviors. The classroom evaluation examined the platform as an integrated teaching environment rather than isolating this component.

#### 2.1.2 Service deployment and training: synergy between large AI models and multimodality

In the platform’s service deployment and training architecture, core capabilities rely on large language models (LLMs), retrieval augmentation, model-management modules, and multimodal collaborative reasoning to provide instructional and exercise-guidance support. During the pilot period, the full DeepSeek-R1 model served as the primary reasoning LLM through the university-approved externally hosted interface (29). The modular architecture also accommodates alternative open-source models when institutional requirements concerning privacy, availability, latency, or cost change. At its core, the LLM + RAG engine combines retrieval from the proprietary knowledge architecture with natural-language generation to produce domain-relevant information for university physical education. The system is evaluated through architecture documentation, representative application scenarios, and classroom outcomes.

To continuously improve answer quality during real-world use, the platform adopts a human-in-the-loop feedback and iterative refinement workflow. Students can report responses that appear incorrect, unclear, or questionable, and physical-education instructors review representative outputs, provide corrections, and update prompt templates, retrieval rules, response constraints, and model-management settings. Interaction information is handled under authentication and de-identification rules.

The model and service layers are designed for deployment flexibility. Domain adaptation is supported through prompt engineering, retrieval rules, model-management procedures, response constraints, teacher review, and the fine-tuning and evaluation modules represented in the system architecture. The reported deployment concentrated on the university-approved configuration and institutional knowledge resources. Table 1 summarizes the data-source categories and processing procedures underpinning this configuration.

**Table 1:** Data sources and processing procedures for domain knowledge resources.

Source category	Access channel	Domain coverage	Processing procedure	Governance
University-library electronic holdings	Institutional library gateway	Physical education, exercise science, fitness, sport psychology, injury prevention, and health management	Normalization, segmentation, metadata retention, retrieval indexing	Licensed access; source provenance
Physical-education teaching resources	Authorized departmental materials	Course content, sport rules, skill learning, teaching cases, and fitness guidance	Format normalization, thematic tagging, instructor review	Role-based access; version control
Campus operational information	Authorized university systems	Course, fitness-test, competition, venue, and service information	Field validation, de-identification, operational-database mapping	Data minimization; permission control

The table summarizes the source categories and processing procedures documented for the reported deployment.

Multimodal collaborative reasoning forms the second pillar, supporting the joint processing of textual commands, sketch annotations, and somatosensory data to generate comprehensive outputs such as tactical-route visualization, technique-learning cues, and exercise-guidance suggestions. To maintain professional fidelity and timeliness in university PE contexts, the platform prioritizes retrieval-grounded prompting, rule-based constraints, and continuous knowledge-base updating, to help outputs remain aligned with verified evidence and institutional teaching standards. In addition, the human-in-the-loop correction workflow provides a practical safeguard against domain-specific errors, enabling the system to iteratively refine prompts, retrieval policies, and safety constraints without requiring frequent heavy-weight model retraining.

The retrieval layer combines semantic and keyword-based retrieval with metadata filtering, while multimodal encoders support textual, image, and sketch resources (30–33).

#### 2.1.3 Data storage, operations, and maintenance: data security and collaborative governance

In terms of data storage and operations, the platform adopts a multi-tier security framework and a collaborative management model to support privacy, compliance, and efficiency. A layered data management system separates student identifiers from professional and analytical databases and uses authenticated access, role-based permissions, data minimization, and de-identification. Federated learning and differential privacy provide privacy-preserving routes for future cross-institutional collaboration (34, 35).

The collaborative operating model integrates contributions from universities, enterprises, and government agencies. Universities provide practical scenarios and user feedback; enterprises focus on technology development and interface optimization; and government institutions establish data-security and ethical guidelines to ensure lawful and transparent operation. For system reliability, the architecture supports access, load, and audit monitoring together with redundant storage, enabling routine operational oversight.

Overall, the platform’s architecture aligns advanced AI technologies with the pedagogical and administrative needs of sports education, supporting coordinated improvements in teaching, training, and management. Its intelligent lesson-preparation and personalized training systems are intended to enhance teaching precision and exercise guidance, while gamified and intelligent scheduling modules can support student engagement and participation. Meanwhile, the emphasis on data security and multi-actor collaboration supports responsible data use and coordinated governance among universities, technical providers, and public authorities.

Future development will deepen the application of digital-twin technology in professional databases, improve multimodal alignment for information interaction, and refine structured tagging for personalized customization. These advancements can provide a stronger technical basis for more efficient and adaptive physical education and should proceed through staged evaluation of technical performance, educational value, and safety.

### 2.2 Construction of the proprietary knowledge base

As a core innovation in AI-generated systems, Retrieval-Augmented Generation (RAG) integrates external knowledge retrieval with generative modeling to improve the grounding and timeliness of domain-specific knowledge services. Within the university’s digital-intelligent sports platform, conventional generative models often suffer from knowledge lag and factual inconsistencies, whereas the RAG framework is designed to improve domain relevance for key functions—such as exercise-prescription generation and tactical analysis—by dynamically retrieving information from a localized proprietary knowledge base. This architecture relies on the coordinated construction of a knowledge graph, a multimodal repository, and structured databases (10, 21), forming a closed-loop “retrieve-augment-generate” mechanism. The knowledge graph provides semantic mapping of entities and relationships; the repository manages unstructured sports resources; and the databases store dynamic user and performance data. Together, they form the platform’s foundational knowledge system as shown in Figure 2. Retrieval quality and factual accuracy nevertheless require independent evaluation.

#### 2.2.1 Construction of the knowledge graph

The construction of the knowledge graph adopts an iterative “LLM generation–human verification” paradigm. By coupling the semantic understanding capabilities of LLMs with expert validation, it supports more structured representations of domain knowledge, as shown in Figure 3.

The knowledge-graph construction workflow consists of five key stages:

1. Initial construction. Using DeepSeek-R1 as the default reasoning model, the platform semantically parses and de-duplicates sport- and PE-related resources accessed through the university library gateway and authorized departmental materials. Coverage includes university physical education, physical activity and fitness, motor-skill learning, injury prevention and rehabilitation, sport psychology, training theory, and health management. Records are normalized, segmented, tagged with source metadata, and indexed for retrieval. Named-entity recognition and relation extraction are then used to extract core triples for a seed knowledge graph (21, 36).
2. Completion and optimization. To enhance coverage, knowledge-graph embedding methods such as TransE and RotatE support prediction of missing relations (37–39). Candidate triples are filtered through domain rules and assisted expert review, and accepted updates are managed through source provenance and version control.
3. Graph-model fusion. Graph-structured data (entities, relations, and attributes) are retrieved alongside text so that the LLM can use structured semantics during generation. Candidate responses are checked against graph constraints, retrieved evidence, and platform rules, while teacher-validated corrections inform prompt, retrieval, and model-management updates within the human-in-the-loop workflow.
4. Knowledge reasoning. Graph traversal and structured prompting support multi-hop connections among training methods, physiological requirements, recovery, and safety. These reasoning paths provide supporting information for teacher-supervised decisions rather than substitute for professional judgment.
5. Updating and management. New resources enter a staged review process in which provenance, publication date, topic labels, and access permissions are recorded. Approved updates are versioned to support traceability and reproducibility.

Table 1 summarizes the verified source categories, access channels, processing procedures, and governance controls used for the domain knowledge resources.

#### 2.2.2 Construction of the knowledge repository

Grounded in event-group training theory (40), the knowledge-base construction categorizes sports by competitive characteristics and training demands to achieve hierarchical organization and precise knowledge services. Based on the dominant determinants of performance, sports are first divided into two macro categories—capacity-oriented (conditioning) and skill-oriented—and further refined into eight event groups: explosive strength, speed, endurance, difficulty and artistry, accuracy, net/partitioned opposition, co-present opposition, and combat sports.

This taxonomy reveals both cross-group regularities and divergent training objectives. Capacity-oriented groups (e.g., weightlifting, marathon) emphasize physiological modeling and load regulation, whereas skill-oriented groups (e.g., gymnastics, football) require standardized analysis of motor skills and tactical reasoning. Accordingly, the knowledge base balances group commonalities with sport-specific traits, forming a closed-loop logic of classification → integration → adaptation to support intelligent responses to complex sporting scenarios, as shown in Figure 4.

For capacity-oriented event groups, the repository organizes physiological, workload, and recovery knowledge for strength, speed, and endurance activities. It links sport-specific indicators with teacher-supervised planning resources, allowing the retrieval layer to provide explanations and level-appropriate guidance (41–43).

For skill-oriented event groups, the repository organizes movement criteria, common errors, tactical relations, rules, and multimodal teaching resources. Pose-estimation and video-analysis methods provide technical routes for linking movement representations with instructional feedback, while opposition-sport resources encode technical and tactical relations for retrieval and teacher review (3, 23).

Across event groups, resources are organized around physical, technical, tactical, psychological, and sport-intelligence dimensions. Hierarchical skill structures and rule-based relations support progression planning, semantic retrieval, and consistency checking (21).

Cross-event retrieval combines keyword and vector-based search with metadata filtering and multimodal representation. Provenance recording, version control, and instructor or domain-expert review govern new entries (31–33).

#### 2.2.3 Construction of the business database

Centered on the paradigm of the sports digital twin, the operational (business) database is designed to establish a bidirectionally driven data ecosystem that dynamically maps students’ physical-fitness parameters to their exercise behaviors. Through authorized fitness information, optional wearables, edge processing, and analytical services, the architecture outlines a closed loop from data acquisition and feature extraction to model updating (8, 44, 45). Its purpose is to transcend the static-storage limitations of traditional databases: through dynamic, incremental modeling, it is intended to explore latent relationships between health and behavior and provide real-time data support for personalized sports services.

To make the digital twin specifically suitable for university PE, the twin state is anchored to educationally meaningful indicators and constraints: (i) campus fitness-test and health-promotion metrics (e.g., BMI, cardiorespiratory fitness proxies, strength/endurance benchmarks), (ii) course schedules and participation contexts (class sessions, extracurricular activity, and event involvement), and (iii) individual safety boundaries derived from prior records and teacher screening. Rather than optimizing elite-performance peaks, the proposed twin would be used to support safe progression and sustained participation by tracking workload–recovery balance, generating level-appropriate prescriptions, and providing interpretable explanations (why a plan is recommended, what to adjust, and how to monitor). It also bridges motivational design: verified activity states could trigger milestone achievements, supporting a potential closed loop of monitoring → guidance → participation reinforcement → re-evaluation.

Architecturally, the database is organized into three tiers—data sensing, virtual mapping, and model iteration. The data-sensing tier can receive authorized fitness, course, movement, and optional wearable information and apply preprocessing before storage. The virtual-mapping tier links user profiles, movement behavior, and dynamic associations in an operational datastore. The model-iteration tier provides an architecture for updating recommendation rules as new validated data become available.

Dynamic linkage between fitness and behavior data is supported through time-series similarity, forecasting, and sequential models. Short-term associations can be modeled through recurrent networks, while longer-term change can be examined with forecasting approaches such as Prophet (46). Together, these methods support dynamic association analysis and iterative recommendation updates within the platform architecture.

For privacy protection, the implemented study used matched records followed by deletion of student identifiers and analysis with anonymous codes. Federated learning and differential privacy are incorporated as privacy-preserving routes for cross-campus collaboration without centralizing raw data (34, 35). Detailed cross-institutional configuration information lies outside the scope of the present university deployment report.

Through the sports digital-twin architecture, the database could support three perspectives for future sport-science research: (1) spatiotemporal analysis of short- and long-term change; (2) personalized adaptation within teacher-defined safety boundaries; and (3) aggregated pattern analysis for service planning. These possibilities remain prospective and require validated measurements, longitudinal data, and explicit governance.

Operationally, the implemented platform followed a sequential pathway. Authorized student, course, fitness-test, and institutional resource requests first passed through identity authentication and role-based access control. Direct identifiers were removed or replaced with anonymous codes before analytical use. Structured operational data were stored in the business database, whereas teaching documents and library resources were normalized, segmented, indexed, and retrieved through the institutional access channel. For a user query, the retrieval layer selected relevant domain materials and assembled them with the query as contextual input to the externally hosted DeepSeek-R1 model. The generated response was returned through the student or teacher interface and, for teaching plans, exercise guidance, and competition arrangements, remained subject to instructor review and revision. User feedback and instructor corrections informed subsequent resource maintenance and iterative refinement.

### 2.3 Design of the user module

**Figure 5:**
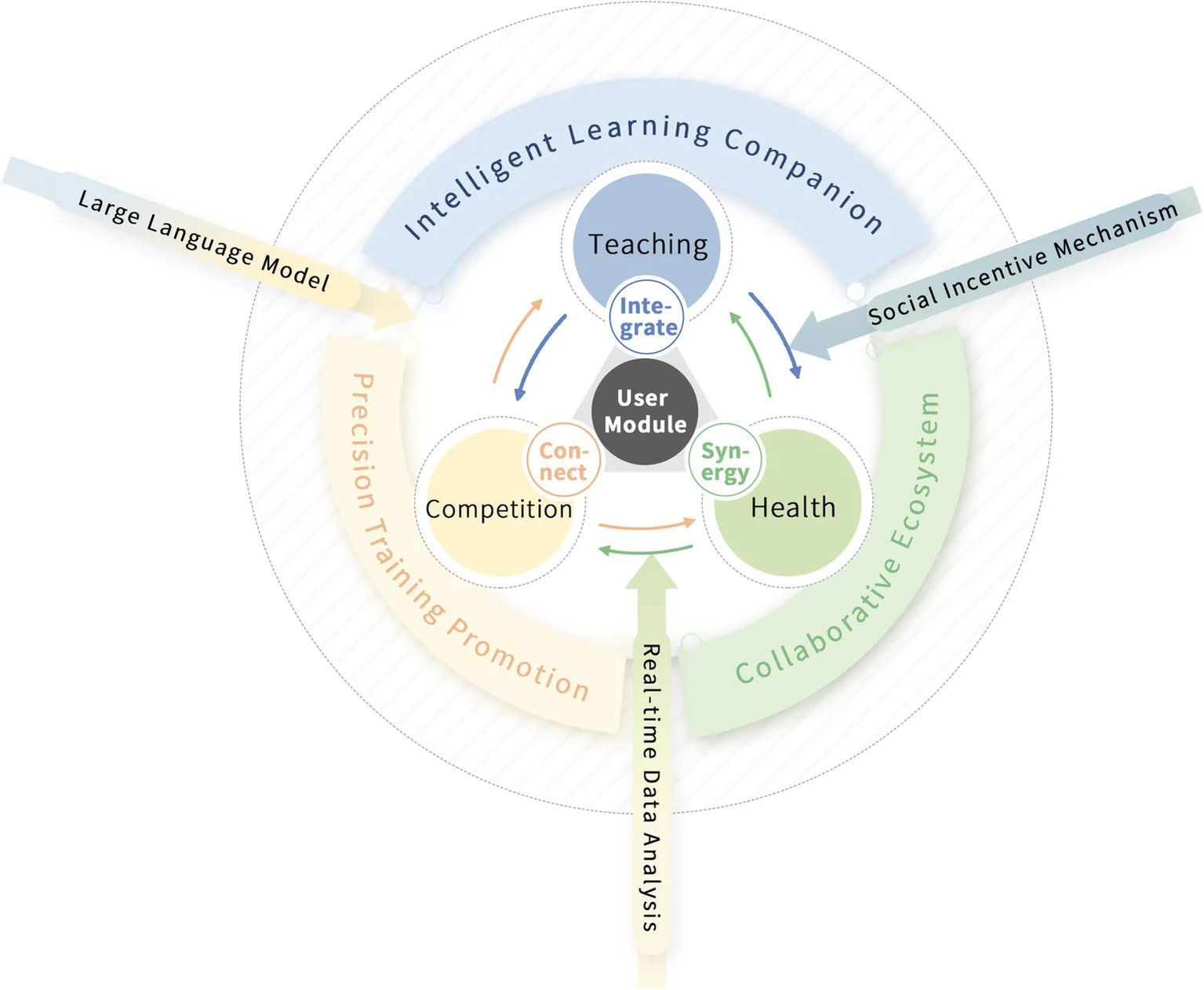
User-module architecture.

The user module adopts a tri-axial architecture of instruction, competition, and health and operates across four functional scenarios—classroom teaching, extracurricular competition, personalized training, and leisure interaction. Together, these components provide integrated support through intelligent learning assistance, personalized training facilitation, and coordinated campus services. Built upon LLMs, real-time data analytics, and social-motivation mechanisms, the system provides an integrated digital service framework for classroom and extracurricular physical-education activities. Figure 5 illustrates this end-to-end framework.

#### 2.3.1 Course instruction module: intelligent study companionship and personalized guidance

The course-instruction module leverages LLMs to create a “smart instructor” system that supports bidirectional interaction both inside and outside the classroom. In theoretical sessions, the module—built on a knowledge graph and a RAG framework—responds to sport-science queries in real time (e.g., the physiological meaning of the anaerobic threshold) and organizes multimodal materials such as anatomical resources and tactics-board examples. In practical sessions, it integrates OpenPose-based motion capture to support assessment of students’ motor skills and provide teacher-reviewed corrective cues—for example, drills addressing insufficient wrist pronation in basketball shooting (47). Transfer learning further enables cross-sport knowledge migration, allowing mechanical principles from badminton swings to inform tennis-serve instruction and supporting cross-sport instructional design (28).

#### 2.3.2 Extracurricular competition module: competition organization and participation incentives

The extracurricular competition module establishes a tiered event system intended to support participation through structured matching and achievement mechanisms. The scheduling subsystem arranges fixtures according to users’ levels and spatiotemporal preferences and synchronizes with venue services. A rules engine organizes officiating precedents and multimodal examples and uses language-model components to retrieve and explain relevant rules (24, 48, 49). Predictive assistance, digital achievement credentials, and officiating support are organized as modular services within the broader architecture, with instructor review and scenario-specific safeguards.

#### 2.3.3 Personalized exercise module: personalized guidance and process support

The personalized exercise module functions as an educational AI coach, generating exercise suggestions based on users’ available profiles, goals, retrieved guidance, and instructor-defined constraints. Recommender methods support content matching (27), and AI-generated exercise programming has begun to be evaluated in collegiate settings (50). Wearable-linked adaptation and nutrition-related guidance are incorporated as modular functions under teacher oversight. The module is intended for educational support and does not replace professional diagnosis or individualized clinical assessment.

#### 2.3.4 Leisure-interaction module: campus participation and social interaction

The leisure-interaction module promotes campus sports culture through an intelligent assistant and team-based collaboration mechanisms. The assistant can generate contextualized interactive content, such as campus orienteering tasks, while team challenges and shared milestones are intended to support participation and social connection. The module also integrates with the venue-reservation system and can recommend feasible time slots. These mechanisms are consistent with autonomy, competence, and relatedness support when they provide meaningful choice, attainable challenge, and social connection (13, 51).

In summary, the platform delivers a triadic service framework—intelligent teaching assistant, mobile exercise coach, and competition-support tool—built on an LLM + RAG architecture. The integrated platform combines resource access, teaching assistance, exercise guidance, organizational support, achievement incentives, digital-twin-related data management, movement assessment, and agent-oriented services. The present study reports the overall architecture and internal application process.

## 3 Application cases of the digitally intelligent platform

### 3.1 Formative campus deployment

**Figure 6:**
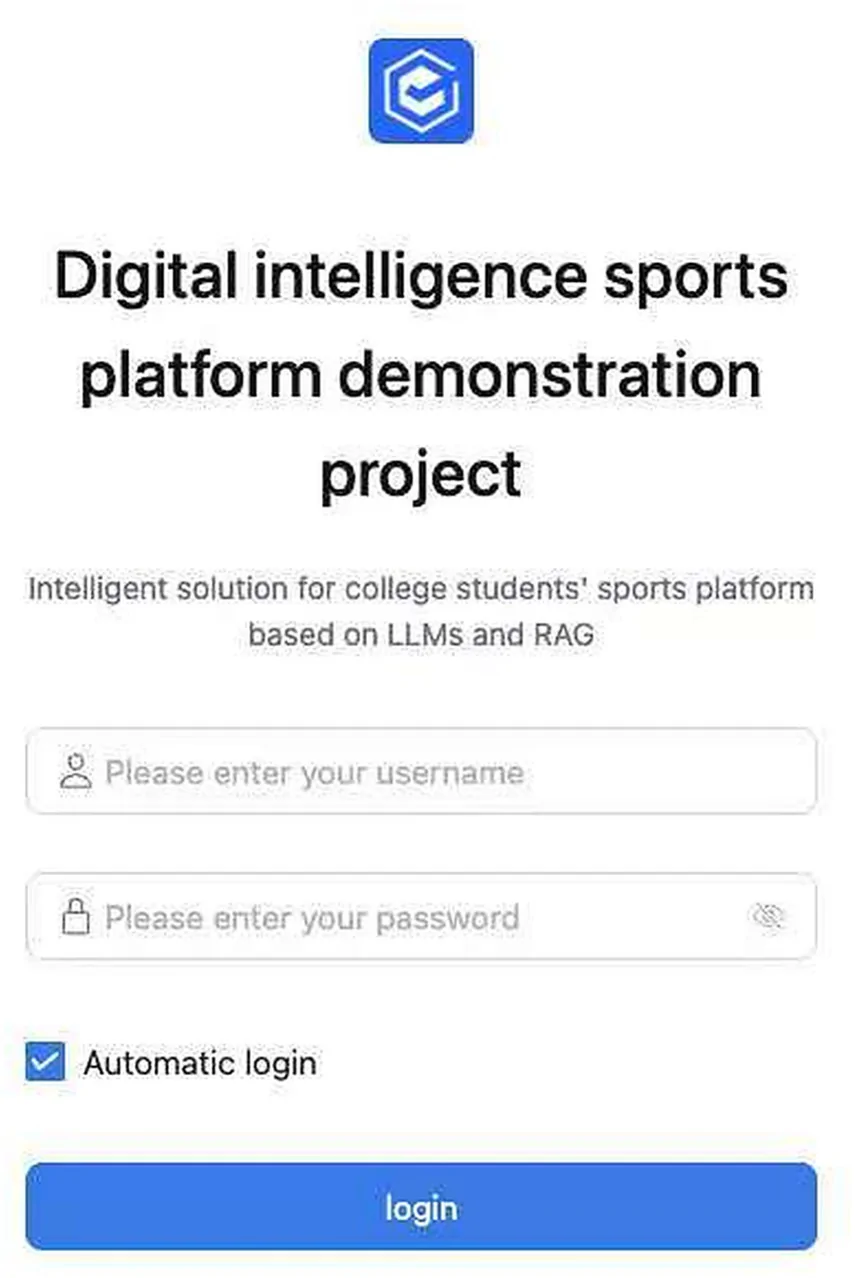
Platform login and user interface.

At a pilot university, a two-month formative deployment involved 428 students and six instructors across table tennis, basketball, and aerobics courses. During the first month, participants accessed the externally hosted full DeepSeek-R1 model directly; during the second month, the same underlying model was embedded in the digital-intelligent platform with institutional retrieval, campus-service integration, and dedicated student and teacher interfaces. This fixed-order deployment was used to examine implementation feasibility and aggregate user perceptions rather than to establish causal effectiveness. The platform was integrated with the university’s office-automation environment and could be accessed by mobile devices, as illustrated in Figure 6.

During the formative deployment, the platform documented operational access through the university’s existing digital environment, use across three PE course types, and teacher- and student-facing scenarios for question answering, lesson preparation, fitness-result interpretation, and competition support. Aggregate questionnaire feedback and informal instructor observations suggested that users valued these integrated functions. The retained summaries are reported descriptively and characterize implementation rather than causal effects.

**Figure 7:**
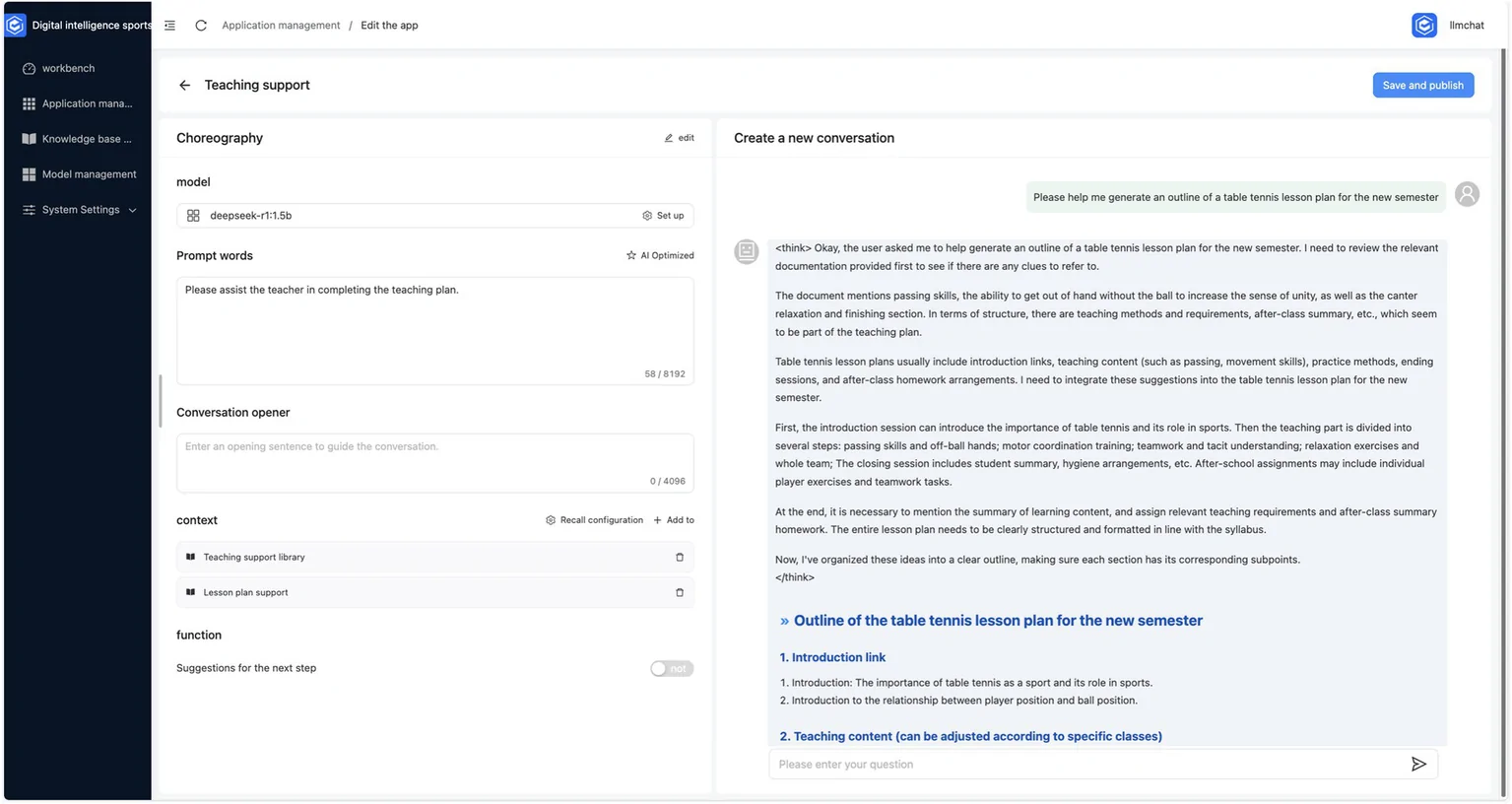
Teacher-facing lesson-preparation interface.

Students used the platform to access sport-related information and teaching resources. Figure 7 shows the lesson-preparation interface used to generate a revisable teaching outline.

**Figure 8:**
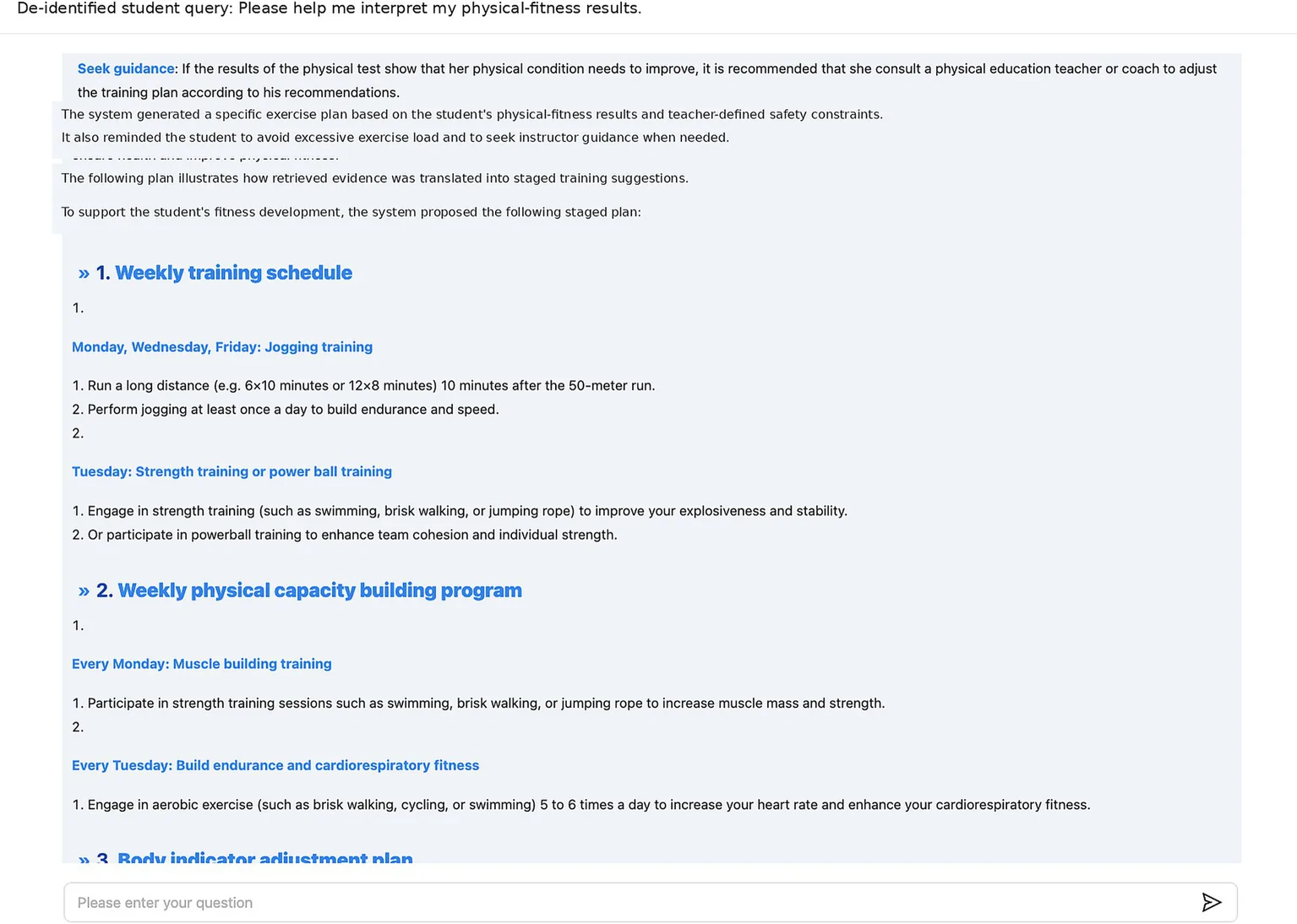
De-identified example of personalized exercise guidance generated during platform use.

Figure 8 presents a de-identified example of personalized exercise guidance generated during actual platform use. The example is included to document the system interface and application process.

**Figure 9:**
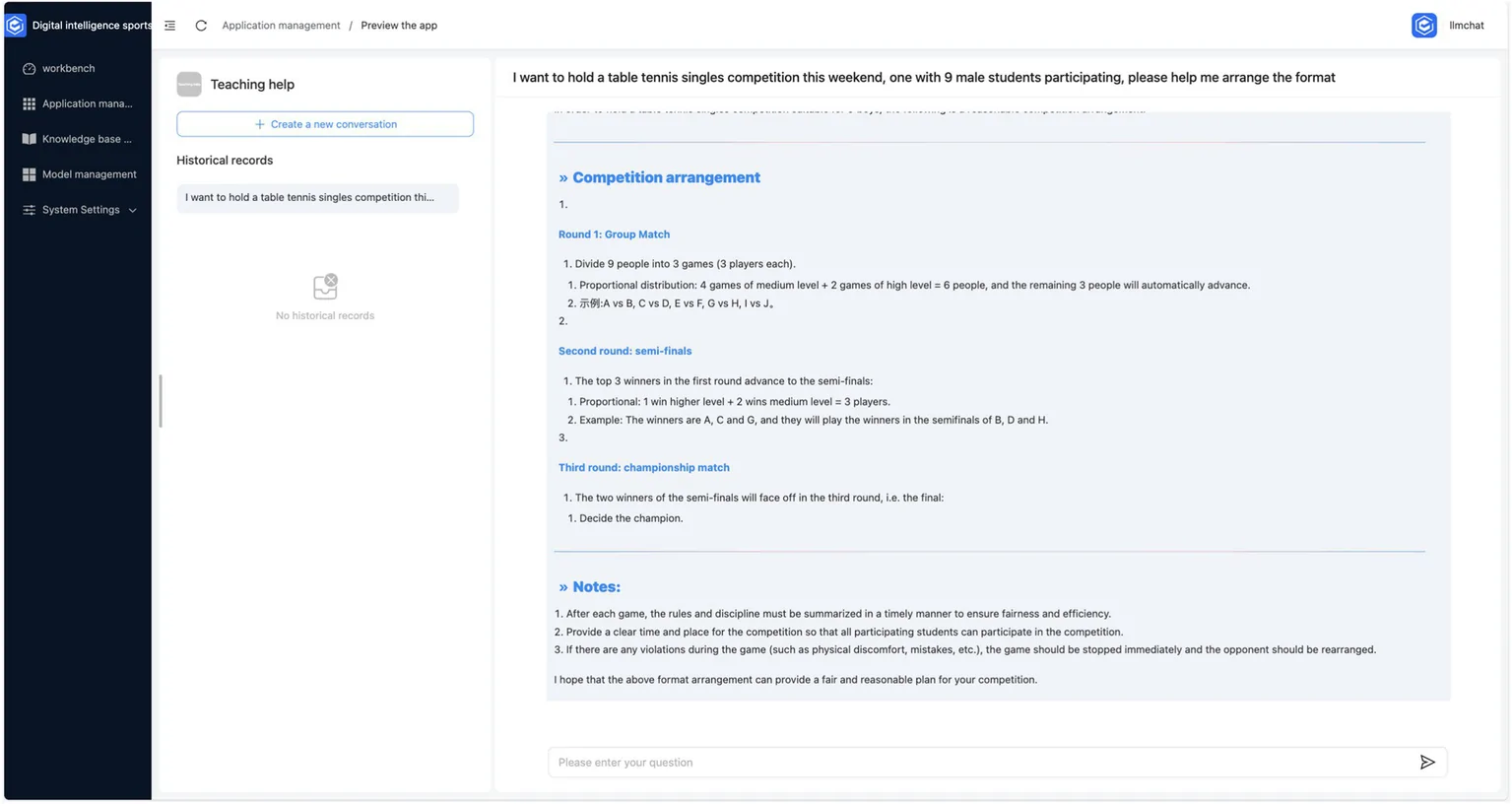
Competition-format and scheduling assistance example.

The platform also supported competition-format generation and organizational assistance. Figure 9 illustrates a teacher-facing example in which the model produced a table-tennis competition arrangement for subsequent instructor review. These examples demonstrate functional integration but do not quantify time savings or organizational efficiency.

Overall, the 428-participant deployment showed that the platform could operate in a real university context and connect model interaction with teaching and campus services. The fixed-order sequence and aggregate nature of the feedback limit causal and module-specific inference. To add a more structured educational evaluation without changing the platform-construction focus, a separate six-week classroom quasi-experiment was conducted.

### 3.2 Six-week classroom quasi-experimental evaluation

#### 3.2.1 Design and participants

An independent, non-randomized, pretest–posttest, intact-class quasi-experiment was conducted from October to December 2025 as an empirical supplement to the platform-construction study. The intervention comprised six weekly physical-education sessions. The sample size was determined by the four complete eligible classes available within the scheduled course implementation. The platform-supported condition included two intact classes (E1, *n* = 44; E2, *n* = 54), and the conventional-teaching condition included two intact classes (C1, *n* = 44; C2, *n* = 54). All four classes were taught by the same instructor. The final paired sample comprised 196 students, with 98 participants in each condition. Intact classes were retained to preserve normal course organization, avoid disruption to teaching schedules, and reduce contamination between instructional conditions; assignment therefore followed existing teaching arrangements rather than individual randomization. The evaluation and reporting strategy was informed by guidance for complex interventions while remaining explicitly exploratory (52).

Pretest and posttest questionnaires were matched by student number before de-identification. Student numbers were then deleted and replaced with anonymous analytical codes. Gender, grade, and major were consistent across all confirmed pairs. Selection-related differences were reduced by including complete classes rather than self-selected volunteers, using the same instructor and instructional schedule, matching pretest and posttest records, adjusting for measured baseline characteristics, and examining class-specific and leave-one-class-out estimates. The classroom data were independent of the 428-participant formative deployment.

#### 3.2.2 Instructional conditions and measures

Both conditions followed the same physical-education schedule and were taught by the same instructor. The platform-supported classes used the digital-intelligent platform for course-related question answering, retrieval of teaching resources, exercise-guidance support, and teacher-mediated learning tasks. The instructor reviewed generated content and retained responsibility for instructional decisions. Control classes received conventional teacher-led instruction without access to the platform during the study period.

Basic psychological need satisfaction and frustration were assessed with a 24-item contextual adaptation of the Basic Psychological Need Satisfaction and Frustration Scale (BPNSFS) (12). Twelve items measured satisfaction and twelve measured frustration across autonomy, competence, and relatedness. Responses ranged from 1 (completely untrue) to 5 (completely true). Need-frustration items were scored in their original direction and were not reverse-coded. Internal-consistency estimates for the two principal scales were high across conditions and assessment points (need satisfaction: *α* = 0.932–0.992, *ω* = 0.940–0.992; need frustration: *α* = 0.908–0.990, *ω* = 0.910–0.990), although these estimates were interpreted alongside the response-pattern audit (Supplementary Table S3). Self-reported physical activity during the previous 7 days was assessed using the International Physical Activity Questionnaire short form (53), from which MET-minutes/week were calculated. Baseline characteristics and outcome distributions are summarized in Table 2.

**Table 2:** Baseline characteristics and outcome measures.

Characteristic	Control	Platform-supported	Standardized difference
Participants, *n*	98	98	—
Female, *n* (%)	39 (39.8)	51 (52.0)	0.25
First-year students, *n* (%)	44 (44.9)	44 (44.9)	0.00
Second-year students, *n* (%)	54 (55.1)	54 (55.1)	0.00
Need satisfaction, mean (SD)	3.77 (0.31)	3.73 (0.42)	−0.13
Need frustration, mean (SD)	2.34 (0.29)	2.16 (0.32)	−0.58
Self-reported activity, median [IQR], MET-min/week	1792 [909, 2,916]	3,173 [2,454, 3,894]	1.01

Values are descriptive; no significance tests were used to compare baseline characteristics. Standardized differences are platform-supported minus control; the activity estimate is based on ln(1 + MET-min/week). IPAQ-derived activity data were available for 97 participants per group at baseline.

Standardized differences indicated similar grade distributions and a small difference in need satisfaction, but differences in sex composition, baseline need frustration, and self-reported physical activity. The corresponding baseline outcome and prespecified covariates were therefore retained in the adjusted models (Table 3).

**Table 3:** Baseline-adjusted classroom outcomes.

Outcome	Adjusted control mean	Adjusted platform mean	Adjusted difference	95% CI	*p*	Standardized difference
Need satisfaction	3.786	4.404	0.618	0.506 to 0.730	<0.001	1.48
Need frustration	2.242	1.589	−0.653	−0.759 to −0.547	<0.001	−1.61
Physical activity (ln-transformed)	—	—	0.202	−0.119 to 0.523	0.216	0.17

Differences are platform-supported minus control at posttest. Models adjusted for the corresponding pretest outcome, gender, grade, and baseline exercise frequency using HC3 robust standard errors. Physical activity was self-reported and analyzed as ln(1 + MET-min/week).

#### 3.2.3 Data quality and statistical analysis

Data-quality screening examined missingness, completion time, within-person BPNSFS variance, and repeated or rule-based response vectors. No questionnaire was automatically removed solely because its response pattern was shared with another participant. Patterned responses were concentrated in the platform-supported condition and are reported as an interpretive limitation. Analyses were conducted in the R statistical environment. For each primary outcome, the posttest score was modeled as the dependent variable, instructional condition was the focal predictor, and the corresponding pretest score, gender, grade, and baseline exercise frequency were entered as covariates. Baseline exercise frequency was represented by its ordered five-category score. HC3 robust standard errors were used because residual diagnostics indicated heteroscedasticity and departures from normality; multicollinearity was not problematic (all variance inflation factors < 2.3). No missing values were imputed, and outcome-specific complete cases were analyzed. The six BPNSFS subdimensions were secondary outcomes with Holm correction. Self-reported physical activity was transformed as ln(1 + MET-min/week) and treated as exploratory because stronger departures from linear-model assumptions persisted after transformation. Because the study included only four intact classes, individual-level inference cannot fully account for class-level dependence; class-specific changes and leave-one-class-out analyses were therefore examined as sensitivity evidence.

### 3.3 Classroom evaluation results

#### 3.3.1 Data-quality and response-pattern audit

**Figure 10:**
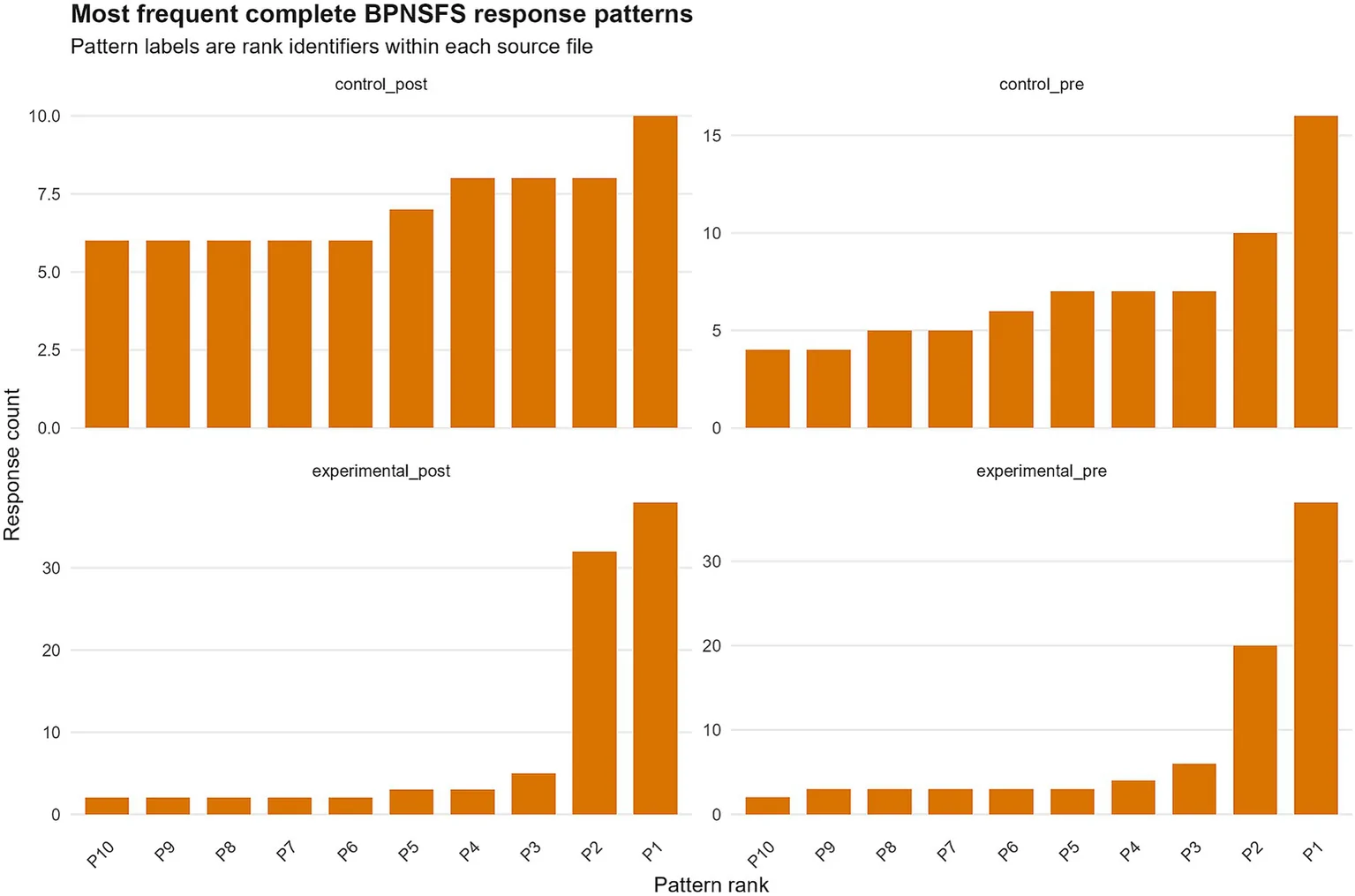
Most frequent complete BPNSFS response patterns by condition and assessment. Pattern labels are within-file rank identifiers; the figure is presented as a transparent response-pattern audit rather than as an exclusion rule.

All 196 participants had confirmed pretest–posttest matches. The response-pattern audit identified 21 and 16 unique complete BPNSFS vectors in the experimental pretest and posttest files, respectively, and 23 and 22 in the corresponding control files. Patterned response structures were more concentrated in the platform-supported group (63 pretest and 70 posttest questionnaires, Supplementary Table S4 and Figure 10). Because response-pattern similarity alone was not used as an invalidity criterion, otherwise complete paired records were retained. The primary estimates were interpreted together with class-specific and leave-one-class-out sensitivity analyses.

#### 3.3.2 Primary outcomes

**Figure 11:**
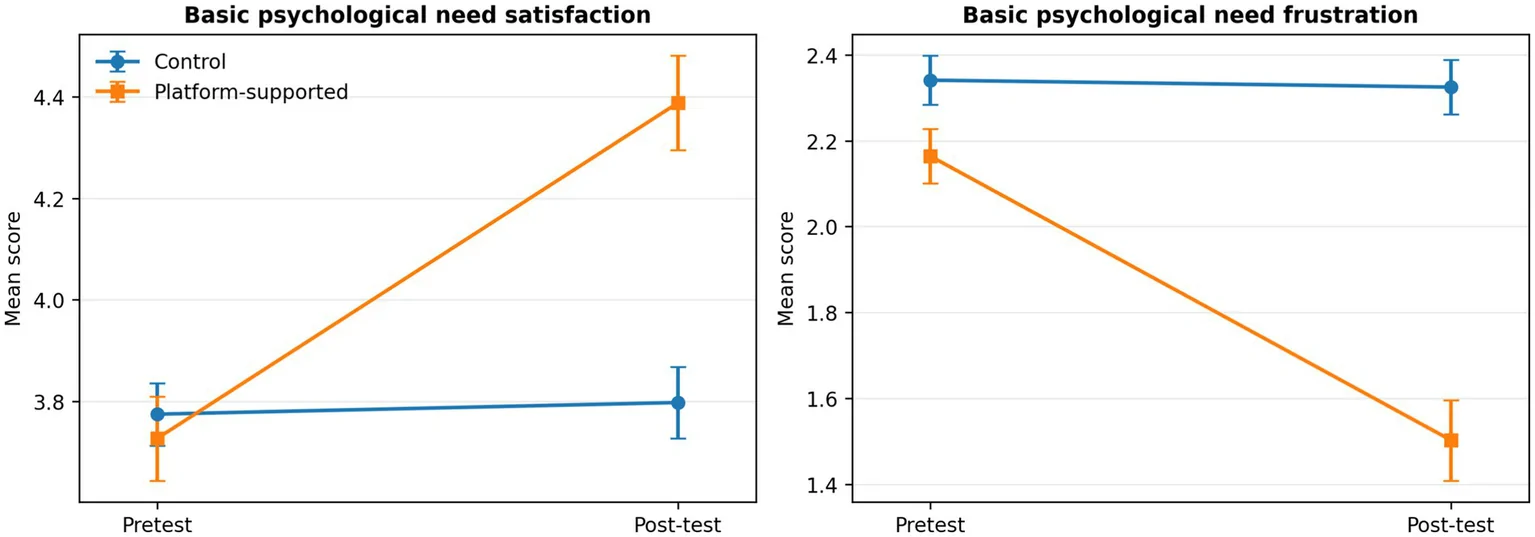
Observed basic psychological need satisfaction and frustration across the six-week classroom evaluation. Error bars represent 95% confidence intervals; adjusted estimates are reported in Table 3.

Outcome-specific complete-case analyses included 195 students for the two primary outcomes because one experimental-group record contained a missing covariate. As shown in Figure 11, observed need satisfaction increased in the platform-supported group and remained nearly unchanged in the control group. The adjusted posttest difference was 0.618 points (95% CI 0.506–0.730, *p* < 0.001), corresponding to a standardized adjusted difference of 1.48. Need frustration decreased in the platform-supported group and remained nearly unchanged in the control group; the adjusted difference was −0.653 points (95% CI −0.759 to −0.547, *p* < 0.001), with a standardized adjusted difference of −1.61.

#### 3.3.3 Secondary and class-sensitivity findings

All six BPNSFS subdimensions favored platform-supported instruction after Holm correction. Adjusted differences ranged from 0.601 to 0.629 for the three satisfaction dimensions and from −0.815 to −0.576 for the three frustration dimensions (Supplementary Table S5). These findings suggest a broad pattern across autonomy, competence, and relatedness rather than a single isolated subdimension.

Changes were directionally consistent in both platform-supported classes. Need-satisfaction mean changes were 0.689 in E1 and 0.637 in E2, compared with 0.038 in C1 and 0.011 in C2. Need-frustration changes were −0.608 in E1 and −0.705 in E2, compared with −0.034 in C1 and −0.002 in C2. Leave-one-class-out estimates remained in the same direction (Supplementary Table S6).

The adjusted between-group difference in self-reported physical activity was not statistically significant (0.202 on the natural-log scale, 95% CI −0.119 to 0.523, *p* = 0.216). Thus, the six-week study detected more favorable psychological-need experiences but did not provide evidence of a corresponding between-group difference in self-reported activity volume. The corresponding distributions are shown in Supplementary Figure S1.

## 4 Discussion and analysis

### 4.1 Classroom evidence and pedagogical interpretation

The technical architecture and classroom evaluation represent two linked levels of the same educational implementation: the former defines how platform support is delivered, whereas the latter examines how students experience platform-supported physical education. Platform-supported instruction was associated with higher need satisfaction and lower need frustration after adjustment for baseline measures and student characteristics. Within Basic Psychological Need Theory, autonomy concerns meaningful choice and ownership, competence concerns effectiveness and optimal challenge, and relatedness concerns social connection (13, 51). The platform may have supported these experiences by expanding access to explanations, revisable learning resources, individualized guidance, and teacher-mediated feedback. Similar principles have been emphasized in physical-education research on autonomy-supportive teaching (14–17).

These findings should nevertheless be interpreted as adjusted associations rather than causal effects. Students were assigned through existing classes, not individual randomization, and only four classes were included. The large standardized differences should be interpreted alongside the response-pattern audit. Although both experimental classes changed in the same direction and leave-one-class-out estimates were stable, replication with more classes, synchronized data collection, and complementary behavioral measures is necessary.

The absence of a statistically significant difference in self-reported physical activity is also informative. Psychological-need experiences are proximal responses to the learning environment, whereas changes in weekly activity may require longer exposure, repeated extracurricular opportunities, and objective monitoring. The findings should therefore not be interpreted as evidence that the platform increased students’ overall physical-activity volume or physical fitness. They support a bounded claim that platform-supported instruction was associated with more favorable psychological experiences during the six-week period.

### 4.2 Challenges to the stability of intelligent technologies and paths to optimization

RAG performance depends on both retrieval quality and generation behavior. Irrelevant or noisy context can reduce answer quality, while rapidly changing operational data can become outdated if retrieval and caching rules are not carefully managed (20). The platform therefore separates relatively stable institutional knowledge from current user-state data and applies source metadata, access rules, and teacher review. Future evaluations should apply standardized, de-identified benchmarks of latency, retrieval quality, factuality, and evidence faithfulness.

Challenges also persist in multimodal alignment and dynamic updating. Knowledge-graph extraction can miss entities or relations, and image–text alignment may drift across sports, camera conditions, and skill levels (9, 21, 33). Expert-assisted review and domain-specific validation are therefore required before multimodal outputs are used for assessment.

On the privacy front, role-based access, de-identification, data minimization, and auditability should precede more advanced privacy-preserving analytics. Federated learning and differential privacy may support future cross-campus collaboration, but their utility and governance implications require empirical testing (34, 35).

### 4.3 Agent-oriented extensions and controlled tool use

Agent-oriented extensions could connect retrieval, scheduling, data interpretation, and feedback through controlled tool use (54, 55). Because these functions extend beyond the present classroom evaluation, future implementations should remain within the existing authentication, operational-data, and instructor-review layers and should be compared with simpler workflows using task completion, evidence faithfulness, unsafe-output rate, latency, and teacher-correction rate.

### 4.4 Sustainable operation and implementation feedback

Following technical refinement, sustainable operation depends on alignment with real teaching work, clear institutional responsibility, and continuous resource maintenance. Universities provide teaching scenarios and governance; technical teams maintain interfaces and retrieval services; and instructors define pedagogical and safety boundaries. Expansion should therefore proceed through staged evaluation rather than promotional diffusion.

The pilot indicates that low-friction authentication, integration with existing campus services, teacher-editable outputs, and transparent source use are more important to sustained adoption than speculative features. Open interfaces and cross-institutional collaboration may be explored only after privacy, interoperability, and quality-control requirements are met.

A sustainable feedback loop should combine de-identified system logs, embedded user feedback, instructor review, and predefined technical tests. These data should be prospectively retained so that changes in retrieval, prompts, or models can be linked to measurable outcomes such as factuality, latency, failure rate, and user task completion.

Although the platform was developed within a Chinese university, its broader educational relevance lies in the modular combination of locally governed knowledge resources, teacher-mediated AI support, and staged classroom evaluation. Universities in other countries could adapt the same design principles by replacing the institutional corpus, curriculum standards, health guidance, language resources, and data-governance rules with locally appropriate equivalents. Cross-national implementation would require attention to local privacy regulations, digital infrastructure, physical-education curricula, teacher preparation, and cultural differences in student engagement.

In sum, sustainable development requires a shift from broad promotional claims toward traceable implementation, teacher accountability, and architecture-aligned validation.

## 5 Conclusions and prospects

### 5.1 Research conclusions

This study develops a university-oriented digital-intelligent sports platform centered on LLM + RAG services, domain knowledge organization, operational data, and user modules. The technical framework and operational data-flow description clarify how data acquisition, storage, retrieval, reasoning, application delivery, and feedback interact. The 428-participant deployment provides formative implementation evidence, while the independent six-week classroom study indicates favorable changes in students’ proximal psychological experiences during platform-supported instruction. No statistically significant between-group difference was identified for self-reported physical activity. Together, the findings support the feasibility of integrating the platform into university physical education, while larger multi-class and multi-institutional studies are needed to assess longer-term educational and technical performance.

### 5.2 Research limitations

Several limitations should be considered. First, the classroom evaluation was non-randomized and involved four intact classes, although both conditions included two classes taught by the same instructor. With only four clusters, individual-level inferential statistics cannot fully separate class-level dependence from the instructional condition. Second, the main outcomes were self-reported. Response-pattern concentration may have contributed to the high internal-consistency estimates and large standardized differences; replication with independently administered measures is therefore warranted. Third, pretest and posttest collection dates were not fully synchronized between conditions. Fourth, the formative deployment did not prospectively collect standardized usability, user-acceptance, satisfaction, system-performance, or AI-response-quality measures and should therefore be interpreted as preliminary implementation evidence rather than a comprehensive platform evaluation. Fifth, the present evaluation focused primarily on educational implementation and classroom outcomes and did not include standardized request-level technical benchmarks.

### 5.3 Future research directions

Future research should proceed in stages aligned with the current architecture. The first stage should preserve versioned corpora and operational records and evaluate AI response quality through factual accuracy, evidence faithfulness, unsupported-claim rate, unsafe-output rate, latency, availability, failure rate, and teacher-correction rate. The second stage should assess usability and acceptance through validated instruments, student and instructor satisfaction, task-completion time, error rates, and sustained use, together with objective educational indicators such as attendance and standardized skill or fitness measures. Objective physical-activity outcomes should also be interpreted against established public-health recommendations for physical activity and sedentary behavior (56). The third stage may introduce a constrained Adaptive Sports Agent that uses authenticated tools, retrieval-grounded planning, explainable guidance, and instructor approval rather than autonomous high-stakes decision-making.

Virtual scenarios, wearable integration, digital twins, privacy-preserving collaboration, and achievement mechanisms may be developed as modular extensions. Each extension should be connected to existing authentication, operational-data, knowledge, and review components and should be activated only after its data requirements, validation criteria, and safety controls have been specified.

## Data Availability

The original contributions presented in the study are included in the article/Supplementary material, further inquiries can be directed to the corresponding authors.
