# Supplementary material for: Artificial intelligence for university physical education: a data–knowledge synergy digital-intelligent sports platform
Source: Front Public Health. 2026 Sep 8;14:1835590. doi: 10.3389/fpubh.2026.1835590 (PMC13593897; doi:10.3389/fpubh.2026.1835590)
Supplement: Supplementary file 1 [file Data_Sheet_1.docx]

Supplementary Material

# Supplementary Methods S1. Functional implementation and reporting scope

The platform manuscript distinguishes the complete system architecture from the level of evidence that can be publicly reported. The modules listed below were implemented, examined internally, or retained as modular system capabilities. Because the university deployment processed protected student physical and behavioral data, granular request-level logs, sensitive student records, and institution-specific configuration details are not disclosed.

**Table S1. Functional status and reporting scope of principal platform components**

| **Component** | **Role in reported work** | **Evidence and reporting boundary** |
| --- | --- | --- |
| DeepSeek-R1 reasoning interface | Primary model interface during the internal deployment | Documented in the platform architecture and application workflow; request-level logs are protected. |
| Institutional RAG retrieval | Retrieval of authorized university-library and PE resources | Source categories and processing procedures are reported; sensitive corpus and query traces are not disclosed. |
| Lesson preparation and question answering | Teacher- and student-facing platform functions | Illustrated by retained interfaces; granular usage records remain within the university system. |
| Exercise-guidance output | Educational guidance under teacher oversight | A de-identified real-use example is presented in the manuscript; no medical diagnosis is claimed. |
| Competition-format and venue support | Teacher-facing organizational assistance | Illustrated by the platform interface and subject to instructor review. |
| Knowledge graph and repository | Domain knowledge organization and retrieval support | Construction workflow and governance are reported; internal graph audit parameters are not publicly disclosed. |
| Movement analysis and multimodal assessment | Video- and sensor-supported educational assessment | Used or examined within the platform; protected student-level records are not disclosed. |
| Digital-twin and privacy-preserving modules | Data mapping and privacy-aware collaboration | Included in the modular architecture; detailed institution-specific configurations lie outside the public report. |
| Agent-oriented services | Coordinated retrieval, tool use, scheduling, and feedback | Included in the architectural roadmap and subject to authenticated access and teacher approval. |

*Note. Status descriptions refer to the two stages reported in the revised manuscript and to the disclosure boundaries imposed by institutional privacy and security requirements.*

# Supplementary Methods S2. Classroom evaluation and scoring

The independent classroom evaluation included four intact classes taught by the same instructor. E1 and E2 received platform-supported instruction; C1 and C2 received conventional instruction. Pretest and posttest questionnaires were paired by student number before identifiers were removed and replaced by anonymous analytical codes.

**Table S2. BPNSFS scoring used in the classroom evaluation**

| **Construct** | **Items** | **Scoring** |
| --- | --- | --- |
| Autonomy satisfaction | 1, 7, 13, 19 | Mean of original 1–5 responses |
| Autonomy frustration | 4, 10, 16, 22 | Mean of original 1–5 responses; not reversed |
| Competence satisfaction | 5, 11, 17, 23 | Mean of original 1–5 responses |
| Competence frustration | 2, 8, 14, 20 | Mean of original 1–5 responses; not reversed |
| Relatedness satisfaction | 3, 9, 15, 21 | Mean of original 1–5 responses |
| Relatedness frustration | 6, 12, 18, 24 | Mean of original 1–5 responses; not reversed |
| Need satisfaction | 12 satisfaction items | Mean of original responses |
| Need frustration | 12 frustration items | Mean of original responses; not reversed |

*Note. The exploratory difference score NeedComp was not used as a primary outcome.*

**Table S3. Internal-consistency estimates for the principal BPNSFS scales**

| **Condition and assessment** | **Need satisfaction α** | **Need satisfaction ω** | **Need frustration α** | **Need frustration ω** |
| --- | --- | --- | --- | --- |
| Platform-supported pretest | 0.988 | 0.989 | 0.973 | 0.974 |
| Platform-supported posttest | 0.992 | 0.992 | 0.990 | 0.990 |
| Control pretest | 0.932 | 0.940 | 0.913 | 0.913 |
| Control posttest | 0.948 | 0.956 | 0.908 | 0.910 |

*Note. α = Cronbach’s alpha; ω = McDonald’s omega total. The high coefficients are interpreted together with the response-pattern audit and are not treated as sufficient evidence of response quality.*

# Supplementary Results S1. Data-quality audit

No participant was automatically excluded because another participant shared the same response vector. Patterned responses were concentrated in the platform-supported classes. The researchers confirmed that questionnaires were completed by the students. Pattern concentration is therefore treated as a measurement-quality limitation rather than evidence of fabricated records. The broad exclusion of any participant flagged at either assessment was not estimable because every platform-supported participant met at least one broad flag; primary analyses consequently used outcome-specific complete cases and emphasized class-specific and leave-one-class-out checks.

**Table S4. Response-pattern audit**

| **File** | **n** | **Unique BPNSFS vectors** | **Patterned responses, n (%)** | **<120 s, n** |
| --- | --- | --- | --- | --- |
| Platform pretest | 98 | 21 | 63 (64.3) | 7 |
| Platform posttest | 98 | 16 | 70 (71.4) | 1 |
| Control pretest | 98 | 23 | 0 (0.0) | 5 |
| Control posttest | 98 | 22 | 0 (0.0) | Not retained |

*Note. Patterned response indicates a rule-based satisfaction/frustration vector. Completion time was unavailable in the recovered control posttest workbook. The corresponding frequency plot is presented as Figure 10 in the main manuscript.*

# Supplementary Results S2. Secondary and sensitivity analyses

**Table S5. Baseline-adjusted BPNSFS subdimension outcomes**

| **Outcome** | **Adjusted difference** | **95% CI** | **Holm-adjusted p** | **Standardized difference** |
| --- | --- | --- | --- | --- |
| Autonomy satisfaction | 0.621 | 0.505 to 0.737 | < 0.001 | 1.43 |
| Autonomy frustration | -0.602 | -0.718 to -0.486 | < 0.001 | -1.39 |
| Competence satisfaction | 0.629 | 0.517 to 0.740 | < 0.001 | 1.45 |
| Competence frustration | -0.815 | -0.913 to -0.718 | < 0.001 | -1.99 |
| Relatedness satisfaction | 0.601 | 0.479 to 0.724 | < 0.001 | 1.44 |
| Relatedness frustration | -0.576 | -0.708 to -0.444 | < 0.001 | -1.29 |

*Note. Models adjusted for baseline outcome, gender, grade, and baseline exercise frequency with HC3 robust standard errors.*

**Table S6. Class-specific change and leave-one-class-out results**

| **Outcome** | **E1** | **E2** | **C1** | **C2** | **Leave-one-class-out range** |
| --- | --- | --- | --- | --- | --- |
| Need satisfaction | 0.689 | 0.637 | 0.038 | 0.011 | 0.592 to 0.656 |
| Need frustration | -0.608 | -0.705 | -0.034 | -0.002 | -0.703 to -0.552 |

*Note. Values for E1–C2 are mean pre/post changes. The final column is the range of baseline-adjusted platform-minus-control differences after excluding one class in turn.*

Figure S1. Distribution of IPAQ-derived self-reported physical activity by condition and assessment.
